# A comparison of termite assemblages from West African savannah and forest ecosystems using morphological and molecular markers

**DOI:** 10.1371/journal.pone.0216986

**Published:** 2019-06-05

**Authors:** Janine Schyra, Jean Norbert B. K. Gbenyedji, Judith Korb

**Affiliations:** 1 Behavioral Biology, University of Osnabrueck, Osnabrueck, Germany; 2 Laboratoire d’Entomologie Appliquée, Département de Zoologie et de Biologie Animale, Université de Lomé, Togo; 3 Evolutionary Biology and Ecology, University of Freiburg, Freiburg, Germany; Universidad de Sevilla, SPAIN

## Abstract

Termites (Isoptera) are important ecosystem engineers of tropical ecosystems. However, they are notoriously difficult to identify, which hinders ecological research. To overcome these problems, we comparatively studied termite assemblages in the two major West African ecosystems, savannah and forest, both under natural settings and along disturbance gradients. We identified all species using morphological as well as molecular markers. We hypothesized species richness to be higher in the forest than the savannah and that it declines with disturbance in both ecosystems. Overall we found more species in the forest than in the savannah. However, alpha diversity per site did not differ between both ecosystems with on average around ten species. For both ecosystems, species diversity did not decrease along the studied disturbance gradient but encounter rates did. For the forest, we did not detect a decline in soil feeding termites and an increase of fungus grower Macrotermitinae with disturbance as some other studies did. Yet, soil feeders were generally rare. Strikingly, the set of morphologically difficult-to-identify Macrotermitinae (*Microtermes* and *Ancistrotermes*) was as high in the forest as in the savannah with little species overlap between both ecosystems. Using phylogenetic community analyses, we found little evidence for strong structuring mechanisms such as environmental filtering or interspecific competition. Most local assemblages did not differ significantly from random assemblages of the regional species pool. Our study is the most comprehensive of its kind. It provides the most reliable termite species list for West Africa that builds the basis for further ecological studies.

## Introduction

Termites are important ecosystem engineers that provide essential ecosystem services in tropical ecosystems. As the main macro-detritivores they influence nutrient flux and food webs and enhance soil fertility, bioturbation and water infiltration rates [1,2,3]. They are prey for many animals, such as ants, spiders, frogs, birds and mammals. For instance, application of the insecticide fibrinol in Madagascar showed how especially termite feeding mammals, such as the lesser hedgehog tenrec *Echinops telfairi*, disappeared after the death of termite colonies [4]. Additionally, large termite mounds (termitaria), such as those of fungus growing Macrotermitinae, provide special micro-habitats for animals and plants alike. They are islands of faunal and floral diversity that increase biodiversity in tropical savannah regions and structure whole ecosystems [5,6,7].

Termite diversity is strongly affected by anthropogenic disturbance. Species richness drastically declines when forests are cleared [8,9] or savannahs are turned into fallows [10]. Along comes also a change in species composition and ‘function’. In forests, several seminal studies have shown that especially soil feeding termites disappear which thrive on soil, rich in organic material [8,11]. Yet our knowledge is still very fragmentary. Studying termite diversity is becoming ever more important in face of intensification of land-use, especially in West Africa where population pressure is increasing [12]. Knowing how termite assemblages are structured and which processes influence assemblage structure in natural, undisturbed habitats builds the basis to understand how termite species assemblages and the associated ecological processes change with anthropogenic disturbance.

Using a standardized approach, we comparatively studied termite assemblages in savannah and forest ecosystems in West-Africa. For each habitat, we compared protected ‘pristine’ sites with disturbed sites that had been un-affected by strong anthropogenic disturbance (i.e., agriculture and clearance) since varying time periods (‘recovery gradient’). Using this approach, we aimed at (i) comparing savannah with forest ecosystems, and (ii) studying how termite assemblages are affected by anthropogenic disturbance in form of intensive land-use to reveal common principles and differences across ecosystems. Based on available literature [8,9,10,11], we hypothesized (i) that the forests will have a higher species richness, especially characterized by many soil feeding species, than the savannah, (ii) that species richness declines with anthropogenic disturbance in both ecosystems, and (iii) that especially soil feeding termites are affected by disturbance in the forest.

To test these hypotheses, we first identified all species occurring in the different assemblages using morphological and molecular markers [13]. A molecular approach using sequence data is necessary to unambiguously identify all termite samples as morphology markers are not sufficient [14, 15]. To obtain first insights into the mechanisms that structure these assemblages, we additionally applied phylogenetic community analyses. They test the composition of our studied communities against communities that are drawn at random from the regional species pool. This approach can provide first hints on the importance of interspecific competition or habitat / environmental filtering in structuring communities [16,17].

## Materials and methods

### Study sites

**Fig 1:**
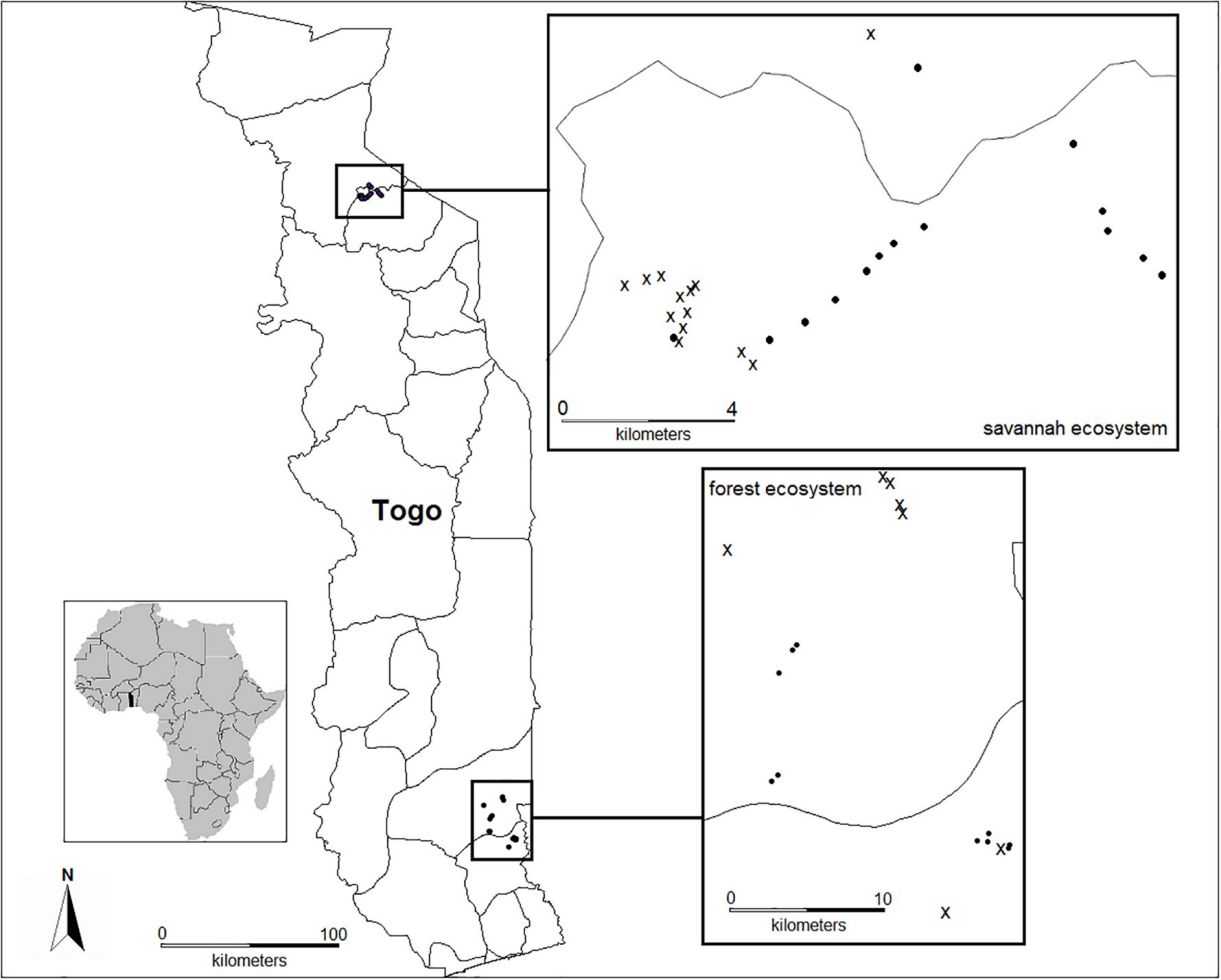
Map of Africa with the location of the two sampling regions savannah and forest in Togo. The savannah in northern Togo with the distribution of the 14 protected sampling sites (Park) and 13 fallows and the forest in the south east of Togo with 10 protected forest sites and seven teak plantation sites. ● = protected sites; **x** = disturbed sites.

As a savannah ecosystem, we investigated termite assemblages in the relatively natural Oti-Kéran National Park (West Africa; 10°17’ to 10°08’ N; 0°28’ to 0°51’ E; Fig 1) in Togo, and compared these assemblages communities with those from a previous study of anthropogenically disturbed habitats (fallows) in the same region [13]. Permissions for the field work were issued by the ‘*Ministere de l’Environement et des Ressources Forestieres*, *Direction Generale de l‘Odef’*, Lomé, Togo. The Oti-Kéran National Park is situated in northern Togo, representing a typical West African savannah, lying in the center of the West Sudanian biome (mean annual precipitation: 1100 mm and mean annual temperature: 28°C, range: 17°C to 39°C; Worldclim database). The park was established in 1950 with an original surface area of 163,640 ha. Since then it has undergone several changes due to socio-political conflicts that reached a climax in the 1990s when the local population invaded the protected area and there was widespread destruction of floral and faunal diversity [12]. In 1999, the government reformed the park boundaries, resulting in a drastic reduction of the park’s surface to 69,000 ha [12]. Today, fields and villages are distributed along the boundaries of the park, but an encroachment of fields and villages inside the protected area can be noticed [18]. Our ‘protected’ study sites were located in areas which were not obviously affected by such human influences. The vegetation of the protected sites consisted of Sudanean savannah. Our study sites consisted of medium-dense shrub savannah characterized by *Crossopteryx febrifuga*, *Vitellaria paradoxa*, *Pilliostigma thonningii*, *Afzelia africana*, *Combretum spp*. and *Terminalia spp*. (see S1 Fig). The disturbed fallow sites had more variable vegetation. Depending on the time since they were last cultivated, they ranged from open fields via grassy savannahs to medium-dense shrub savannah (S1 Fig).

Our protected forest sites were located in the south east of Togo, in the Reserve de Faune de Togodo (6°40’ to 6°50’ N and 1°20’to 1°40’ E, Fig 1). This reserve covers an area of about 25,500 ha and has an equatorial climate, with 1000 to 1300 mm of rainfall per year and mean annual temperatures of 27°C (range: 25°C to 29°C) [19]. It comprises semi-deciduous forests with stratified vegetation, consisting of an often closed tree canopy and an evergreen woody undergrowth (S2 Fig). Overall, the forest is dense and the tree cover closed. It harbours more than 100 tree species; the most dominant species are *Drypetes floribunda*, *Ceiba pentandra*, and *Antiaris africana* (J.N. Gbenyedji, unpubl. data).

Sampling of disturbed sites was carried out around the reserves boundaries in anthropogenically established forests, teak plantations. Depending on the age since establishment, it ranges from a very open area with teak samplings into a forest with tall trees (S2 Fig). Yet, it never has a woody understory and is characterised by the even spaced teak trees (S2 Fig).

Similar to fallows in the savannah, teak plantations can be regarded to reflect anthropogenic disturbance gradients for forests in this region. Disturbance is maximal, when teak plantations are established as the original vegetation is completely removed (corresponding to fields in the savannah region). Disturbance declines with increasing time since start of the plantation (corresponding to fallow age in the savannah). The comparison suffers from the fact that teak plantations are still largely mono-cultures. Yet, teak plantations are the best equivalent present in Togo, as secondary forests of known age are lacking. Additionally, they present a large part of converted land in equatorial West Africa [19,20], hence, being of fundamental economic and ecological importance.

### Termite sampling

Termites were systematically collected when they were most active. For the savannah, termites were collected during the beginning of the rainy season in 2012 from nine plots in the Oti-Kéran National Park and from seven fallows of age 0, 2, 4, 6, 8, 10 and 12 years in its surroundings. In 2014, we added five new plots for the natural habitat and six plots for the fallows of age 0, 0, 1, 2, 10 and 10 years. ‘Fallow age’ corresponds to the time since the plots were last used in agriculture and thus the time since last anthropogenic disturbance. The sampling regime was constrained by the availability of fallows with known age.

For the forest, we sampled ten sites in September and in December 2012 in the Reserve de Faune de Togodo. Sampling of disturbed sites was carried out in September/October and December 2012. Similar to the study in the savannah, seven teak plantations of ages 0, 2, 4, 6, 8, 10 and 12 years were sampled. The age of the teak plantation also corresponds to the time since last anthropogenic disturbance/interference.

Sampling was done using a standardized belt transect protocol first developed for sampling termites in forests [21] and then adapted to savannahs [14]. In short, the protocol consists of a thorough search of dead plant material on the ground, on and in trees and mounds as well as soil sampling to assess termite diversity [21]. For both regions, plot size was one hectare with three transects arbitrarily located within each plot. Corresponding to the established protocols, in the savannah each transect measured 50 m x 2 m, divided into ten 5 m x 2 m sections, while those for the forest were 100 m x 2 m with twenty 5 m x 2 m sections. Each transect section was searched by a trained person systematically for termites for 15 minutes in the savannah, and 30 minutes in the forest. Additionally, we sampled eight soil scrapes per transect section measuring 15 cm x 15 cm x 10 cm. For analyses, each transect was treated as one replicate, so that we had three replicates that characterized a site [22].

All encountered termites were stored in 99% ethanol for subsequent molecular analyses.

As in the former studies [13,14,21], we chose a plot size of one hectare because the foraging ranges of termite colonies is within 100 m [23] and hence one hectare represents the local scale where interactions between colonies occur, i.e. it reflects the Darwin-Hutchinson-Zone, which is most relevant to study assembly of local assemblages [24].

### Identification and phylogenetic analyses

All samples were identified to the species level: First, samples containing soldiers were identified to the genus and species level using the keys by Webb [25], Sands [26], Bouillon & Mathot [27], Harris [28], Grassé [29], Pearce *et al*. [30] and Sands [31,32] and then confirmed by molecular sequence analyses (see below). For samples with workers only, morphological identification was impossible, they were genetically analysed (see below). We followed the anatomical criteria by Donovan *et al*. [33] to assign the feeding group to each sample. The presence / absence of each species within each plot was recorded as well as the encounter rate (i.e., the number of samples per species and plot), which is used as a surrogate of species abundance [34].

**Fig 2:**
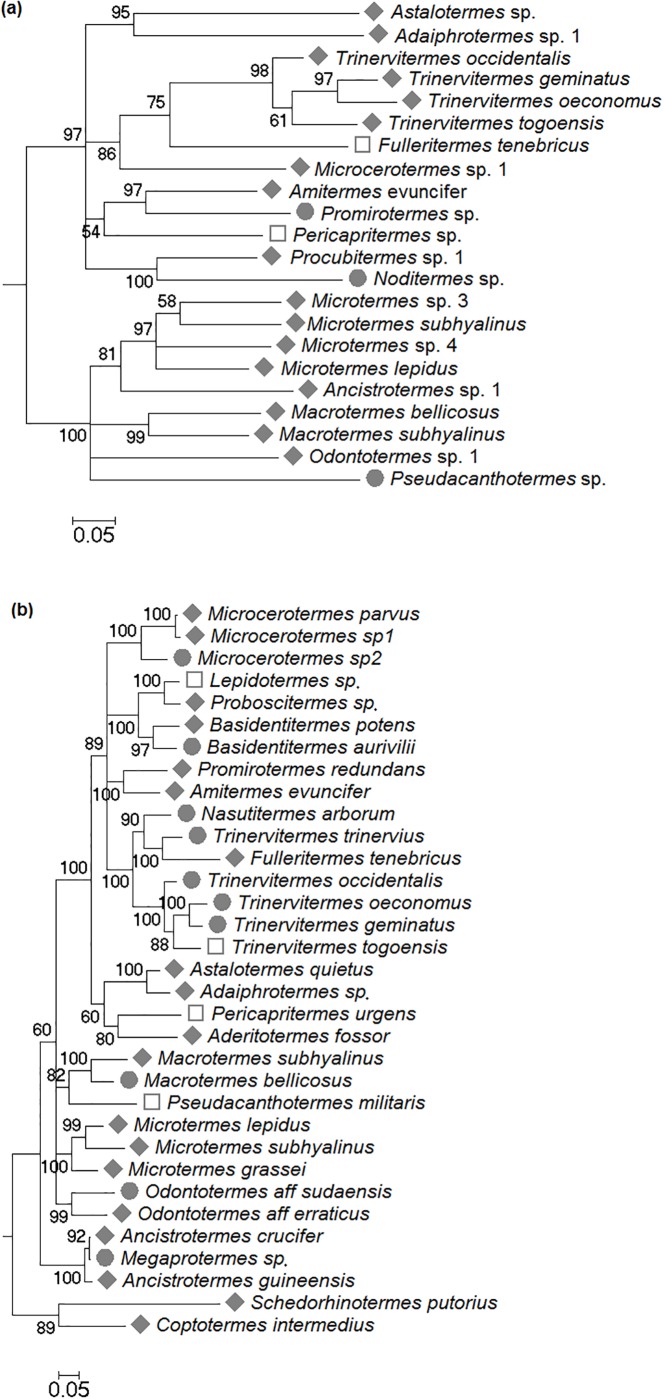
Input Bayesian phylogeny of the (a) savannah and (b) tropical forest ecosystems for the program Phylocom based on the gene *cytochrome oxidase II*. Occurrence of species: ♦ = in both regimes; ● = protected only; □ = disturbed only.

Termites are often difficult to identify by morphological traits alone as they commonly lack species-specific morphological traits (especially fungus-growing species), and contain cryptic species [14,15]. To allow unambiguous species identification, we have applied a molecular approach in a former study that investigated the effect of disturbance in the savannah [13]. There, we sequenced fragments of the genes *cytochrome oxidase I* (*COI)*, *cytochrome oxidase II* (*COII*) and *12S* and applied three phylogenetic approaches (Bayesian, maximum parsimony (MP) and Maximum Likelihood (ML)) to infer species [13]. We also followed the same approach and analyses for our forest dataset. Similar as in other termite studies [14,35], *COII* was the best markers as it amplified well and resulted in well resolved trees (Fig 2). For both *COI* and *12S*, amplification often failed and especially *12S* was too conserved so that it often did not resolve species but only genera in the inferred trees. These problems have also been encountered in other termite studies [14,35].

### Species delimitation analyses

Complementary, we also used single- and multi-rate poisson tree processes [36,37], as implement in the software mPTP [37] v. 0.2.4., to confirm how many and which species occurred in each ecosystem. Since this software cannot cope with polytomies in a tree (which we got in the former analyses; Fig 2) and the tree needs to be rooted with an outgroup not too distantly related, we did additional ML tree inferences, one analysis for each ecosystem.

For the Savannah dataset, we choose 147 *COII* sequences of high quality from the savannah samples (all Termitidae). As outgroup we selected *Coptotermes intermedius* (Rhinotermitidae) to root the tree.

For the Forest dataset, we selected 183 high quality sequences from the forest and used *Coptotermes intermedius* and *Schedorhinotermes putorius* as outgroups to root the trees. (Note, we had to exclude the single available sequence of *Megaprotermes sp*. because of bad sequence quality. Hence this species is missing in this analysis). All sequences have been uploaded in Genbank (Tables with accession numbers: savannah: S1 Table, forest: S2 Table) Both alignments are also provided as Supplementary material in Fasta format (savannah: S1 Appendix, forest: S2 Appendix).

For both datasets, we generated multiple sequence alignments with MAFFT 7.313 [38] using the L-INS-i algorithm. For phylogenetic tree inference, we performed for each dataset ten separate ML tree inferences using the software IQ-TREE [39] version 1.6.8. with a random starting tree and estimating the best-fitting nucleotide model using Modelfinder [40] in IQ-TREE beforehand (-mMFP). For model selection, we chose the corrected AKAIKE information criterium (AICc) [41]. We choose from the ten inferred ML trees, the tree with the best log-likelihood value as best tree. The best estimated model was TIM2+F+R3 for the Savannah and TN+F+I+G4 for the Forest.

Subsequently, we inferred for each dataset 1,000 non-parametric bootstrap replicates (-bo) with IQ-TREE and mapped these onto the ML tree with the best log-likelihood. We checked for bootstrap convergence *a posteriori* according to Pattengale et al. [42] and as implemented in in RAxML (v.8.2.11) [43] (settings:”autoMRE”–B 0.03 and–bootstop-perms 10,000). Bootstrap convergence was fulfilled for both datasets after 600 bootstrap replicates.

The resulting trees (savannah: S3 Fig, forest: S4 Fig) revealed the same sample clusters and were topological identical to trees from the Bayesian analyses (Fig 2) except that the polytomies of the latter were resolved.

We then used the best ML trees, rooted with *Coptotermes intermedius* for the Savannah dataset and rooted with *Coptotermes intermedius* plus *Schedorhinotermes putorius* for the Forest dataset, as input for species delimitation using mptp [37], analyzing each dataset separately.

We first estimated the minimal branch length based on the alignments (—minbr auto). With implemented maximum-likelihood heuristics, we performed species delimitation analyses with a random seed considering the minimal branch length. On the Savannah dataset, we applied the multi-rate assumption (—multi). For the Forest dataset, we expected many ‘singleton’ species (i.e., only one sample per species, according to the tree inference approach). According to P. Kapli (pers. com.), the multi-rate model behaves suboptimal in such cases and the single-rate ptp estimate is recommended as it sets threshold on well sampled species. Therefore, we used the single-rate approach for delimiting species of the Forest dataset. We assessed branch support by performing four MCMC chains for each dataset. For each run, a random starting point was chosen for 100 million generations, sampling every 100000^th^ generation. We discarded the first five million generations as burn-in.

### Phylogenetic community structure analyses

We analysed the local assemblage structure with PHYLOCOM 4.2 [44]. As input tree for the phylogenetic structure analyses we used the *COII* gene tree inferred with the Bayesian approach. This tree was pruned prior to analysis so as to have only species of the regional species pool included and only one representative sample per species in the tree. This representative was the sequence with the highest quality values for each base (maximum value of 61, multiplied by ten) as defined in Chromas 2.4.4 (1998–2016, Technilysium Pty Ltd).

We calculated the net relatedness index (NRI) that measures whether locally co-occurring species are phylogenetically more / less closely related than expected by chance. It uses phylogenetic branch length to measure the distance between each sample to every other terminal sample in the phylogenetic tree, and hence the degree of overall clustering [44]. The NRI is the difference between the mean phylogenetic distance (MPD) of the tested local community and the MPD of the total community (regional) divided by the standard deviation of the latter. High positive values indicate clustering (high similarity); low negative values overdispersion (low similarity) [45]. We tested whether our data significantly deviated from 999 random communities derived from null models using the independent swap algorithm on presence / absence data [46,47]. The swap algorithm creates swapped versions of the sample / species matrix and constrains row (species) and column (species’ presence or absence) totals to match the original matrix. The regional species pool consisted of all species from all studied localities. As suggested by Webb et al. [44], we used two-tailed significance tests based on the ranks that describe how often the values for the observed community were lower or higher than the random communities. With 999 randomizations, ranks equal or higher than 975 or equal and lower than 25 are significant at *P* ≤ 0.05 [45].

### Similarity between fallows

We quantified the compositional similarity (ß-diversity) between all localities using the Bray-Curtis sample similarity index [48], which was calculated in EstimateS version 8.2.0 [49]. It ranges from 0 to 1, with low values indicating low similarity and high values the reverse. In analogy to this, we assessed the pairwise phylogenetic similarities between sites using phylo-ß-diversity, which measures how phylogenetic relatedness changes between sites [50]. Here high values indicate high phylogenetic similarity and low values low phylogenetic similarity between communities [50]. This PhyloSor index was calculated with the package ‘picante’ in R [51].

### Other statistical analyses

All inferential statistics were done with the statistical package IBM SPSS 16. All tests were two-tailed. Data were tested for assumptions of parametric testing and analyses were done accordingly. For all data, qualitatively the same results (i.e., effects were significant or non- significant) were obtained when testing parametrically or non-parametrically.

## Results

### Diversity

The taxonomy of West African Termite taxonomy faces huge problems as many species could not be reliably identified using morphological markers only. Out of a total of 40 species, 28 species (Table 1) were only possible to unambiguously identify with the help of molecular markers. This included fungus-grower which are known to be notoriously difficult to identify, but also Trinervitermes species for which a species-specific identification key exists [26]. Comparing the phylogenetic inference approach with the species delimitation analyses after Kapli [37], both methods revealed identical results, except for four species in the forest (Table 1, savannah: S5 Fig, S3 Appendix; forest: S6 Fig, S4 Appendix). Huge problems exist for *Ancistrotermes guineensis* where samples build a single cluster in the tree, but were delimitated as 15 species with the delimitation approach. Thus, more work is needed to resolve the taxonomic status of this species. *Ancistrotermes crucifer*, *Nasutitermes arborum*, and *Microtermes subhyalinus* were delimitated as two species each which can be explained by geographic variation and population substructuring. In the following, we treat each of these four cases as a single species as they are currently considered as ‘good’ species in other studies.

**Table 1:** Comparison of the regional species pools for the savannah and tropical forest with presence/absence and total species richness.

		savannah		tropical forest	
	fg	Park	fallows	Forest	Teak pl.
**RHINOTERMITIDAE**					
**Rhinotermitinae**					
*Schedorhinotermes putorius*^1^	I	-	-	X	X
**Coptotermitinae**					
*Coptotermes intermedius*^1^	I	-	-	X	X
**TERMITIDAE**					
**Nasutitermitinae**					
*Trinervitermes trinervius* ^1^^,^^2^	II_g_	-	-	X	-
*Trinervitermes occidentalis*^1^^,^^2^	II_g_	X	X	X	-
*Trinervitermes geminatus*^1^^,^^2^	II_g_	X	X	X	-
*Trinervitermes oeconomus*^1^^,^^2^	II_g_	X	X	X	-
*Trinervitermes togoensis*^1^^,^^2^	II_g_	X	X	-	X
*Fulleritermes tenebricus*^1^	II	-	X	X	X
*Nasutitermes arborum*	II	-	-	X	-
**Macrotermitinae**					
*Microtermes subhyalinus*^(1)^	II_f_	X	X	X	X
*Microtermes lepidus*^1^^,^^2^	II_f_	X	X	X	X
*Microtermes grassei*^1^^,^^2^	II_f_	-	-	X	X
*Microtermes* sp.3^1^^,^^2^	II_f_	X	X	-	-
*Microtermes* sp.4^1^^,^^2^	II_f_	X	X	-	-
*Ancistrotermes crucifer*	II_f_	-	-	X	X
*Ancistrotermes guineensis*	II_f_	-	-	X	X
*Ancistrotermes* sp.1^1^^,^^2^	II_f_	X	X	-	-
*Macrotermes bellicosus*^1^	II_f_	X	X	X	-
*Macrotermes subhyalinus*^1^	II_f_	X	X	X	X
*Odontotermes aff*. *erraticus*^1^^,^^2^	II_f_	-	-	X	X
*Odontotermes aff*. *sudaensis*^1^^,^^2^	II_f_	-	-	X	-
*Odontotermes* sp.1^1^^,^^2^	II_f_	X	X	-	-
*Pseudacanthotermes militaris*^1^	II_f_	X	-	-	X
*Megaprotermes* sp.^1^	II_f_	-	-	X	-
**Apicotermitinae**					
*Astalotermes quietus*^1^^,^^2^	III	X	X	X	X
*Adaiphrotermes* sp^(1)^^,^ ^2^	III	X	X	X	X
*Aderitotermes fossor*^1^^,^^2^	III	-	-	X	X
**Termitinae**					
*Microcerotermes* sp.1^1^^,^^2^	II	X	X	X	X
*Microcerotermes* sp.2^1^^,^^2^	II	-	-	X	-
*Microcerotermes parvus*^1^^,^^2^	II	-	-	X	X
*Basidentitermes potens*^1^^,^^2^	IV	-	-	X	X
*Basidentitermes aurivilli*^1^^,^^2^	IV	-	-	X	-
*Promirotermes redundans*^1^^,^^2^	III	X	-	X	X
*Proboscitermes* sp. ^1^^,^^2^	IV	-	-	X	X
*Amitermes evuncifer*^1^	II	X	X	X	X
*Pericapritermes urgens*^1^^,^^2^	III	-	-	-	X
*Pericapritermes* sp. ^1^^,^^2^	III	-	X	-	-
*Lepidotermes* sp. ^1^^,^^2^	IV	-	-	-	X
*Noditermes* sp. ^1^^,^^2^	IV	X	-	-	-
*Procubitermes* sp. ^1^^,^^2^	III	X	X	-	-
**total species**		**20**	**19**	**29**	**23**

Shown are presence/absence and total numbers of species including feeding groups (fg) for the two ecosystems savannah and tropical forest with its classification into protected and disturbed habitats. The classification of feeding groups follows Donovan *et al*. [33]: I: dead wood-feeders; II: wood-litter feeders (II_g_: grass feeders; II_f_: fungus growers); III: humus feeders; IV: *true* soil feeders.

^1^ species which were identified identically with the phylogenetic inference and the species delimitation approach;

^(1)^ species which were identified identically with both approaches in the savannah but not in forest,

^2^species which were only possible to unambiguously identify with the help of the molecular approaches but not with morphological traits.

#### Savannah

We identified a total of 22 termite species in the savannah, representing the regional species pool, with 20 species in the protected national park (three species unique to this habitat) and 19 species in the sampled fallows (two species unique for this habitat) (Table 1, Fig 2a and 2b, S3–S6 Figs). The park sites and fallows shared 17 species of the regional species pool. All 22 species belonged to the Termitidae. As is typical for African savannahs, the fungus-growing Macrotermitinae dominated under both habitat regimes with nine species in the Park and eight species in the fallows. We found two Apicotermitinae each in the Park and fallows, five Termitinae in the Park and four in the fallows, and four Nasutitermitinae in the Park and five in the fallows (Table 1). Species richness did not increase with fallow age (Spearman-rank correlation: N = 13, P = 0.324).

#### Forest

In the forest, we identified a total of 33 termite species. Thus, the regional species pool was more diverse in the sampled forest sites and teak plantations than in the savannah, although overall species richness per site was not significantly different (Mann-Whitney-U test, Z = -0.012, N = 44, P = 0.990). Twenty nine species were sampled in the natural forests, with 10 species being unique for this protected area, and 23 species in the teak plantations, of which 4 species were unique for this habitat (Table 1). The protected sites and the teak plantations shared 19 termite species and most species belonged to the higher termites, with 10 Macrotermitinae in the forest and eight in the teak plantations, eight Termitinae in the forest and teak plantations each, three Apicotermitinae each in both habitats, and six Nasutitermitinae in the forest and only two in the teak plantations. Additionally, two representatives of the lower termites were sampled in the forest habitat which did not occur in the savannah: one species of the Rhinotermitinae and one species of the Coptotermitinae. Similar to the results in the savannah, species richness did not increase with increasing plantation age (Spearman-rank correlation: N = 7, P = 0.534). As is typical for forests, more soil feeders (feeding group IV) were sampled here compared to the savannah (Table 1).

### Local phylogenetic community structure

#### Savannah

The NRI values, measuring if locally co-occurring termite species are phylogenetically more or less closely related than expected by chance, ranged from -1.05 to 3.28 in the Park and from -0.72 to 4.21 in the fallows. Only a few plots in the Park and fallows showed significant phylogenetic clustering or overdispersion. In the Park, two plots were significantly clustered (Plot I: NRI = 4.46; Plot 1: NRI = 1.99, P < 0.05) and one plot had a significant signal of overdispersion (Plot D: -1.38, P < 0.05). In the fallows three plots showed significant signals of clustering (Plot S: NRI = 2.83; Plot W: NRI = 2.80; Plot 5: NRI = 4.41, P < 0.05).

**Fig 3:**
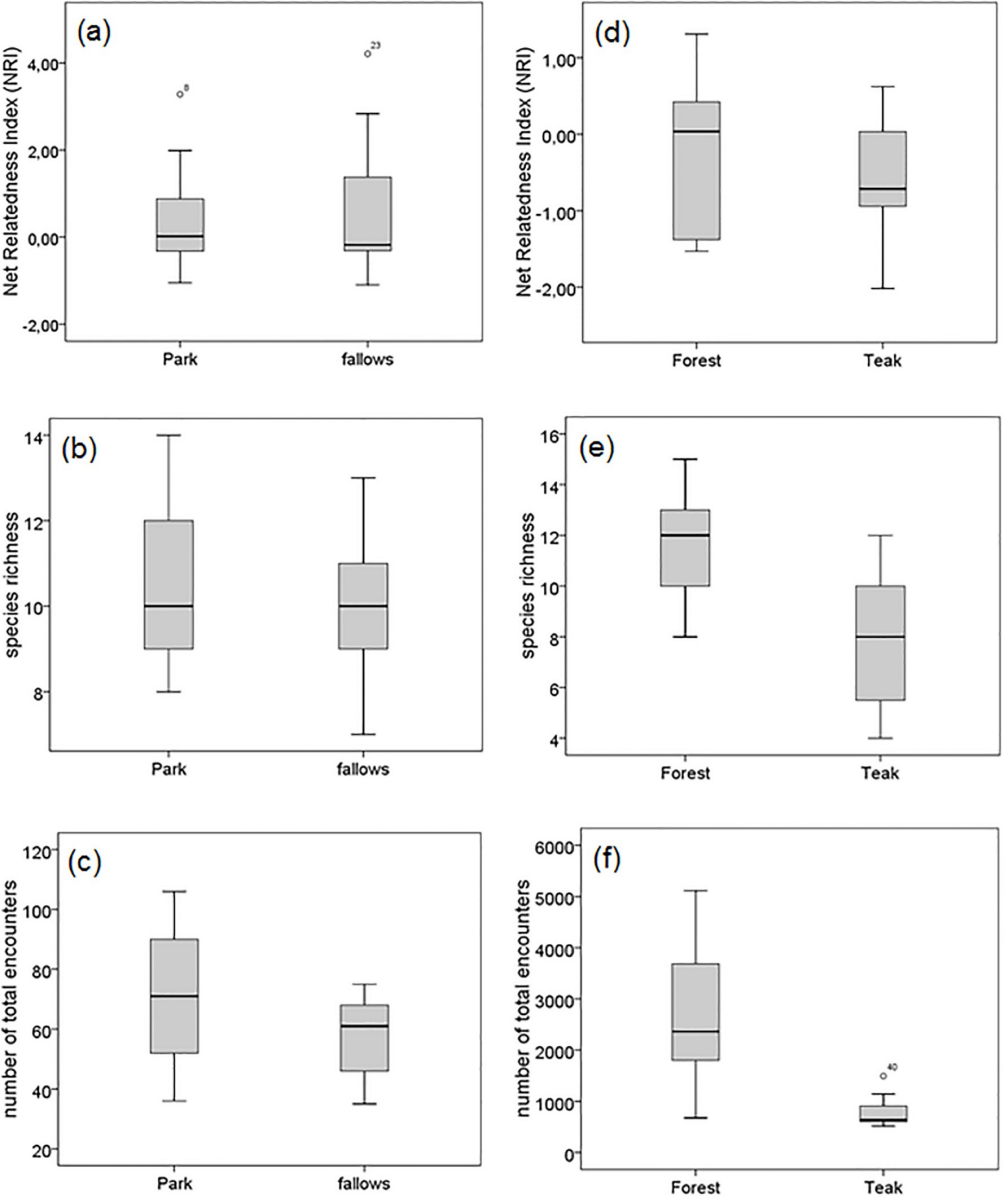
Comparison of (a) net relatedness index (NRI), (b) species richness and (c) number of total encounters between park and fallows in comparison with (d) net relatedness index (NRI), (e) species richness and (f) number of total encounters between forest and teak plantations.

NRI values and species richness did not significantly differ between habitat regimes (Mann-Whitney-U test, NRI: Z = -0.09, N = 27, P = 0.923; species richness: Z = -0.71, N = 27, P = 0.473) (Fig 3a and 3b). The number of total encounters of termites seemed slightly (though not significantly) lower in the fallows than in the Park (Mann-Whitney-U test, Z = -1.52, N = 27, P = 0.126, Fig 3c).

#### Forest

NRI values ranged from -1.52 to 1.3 in the forest and from -2.01 to 0.62 in the teak plantations. NRI values were higher in the savannah than in the forest, but not significantly (Mann-Whitney-U test: Z = -1.53, N = 44, P = 0.126). Similar to the savannah, forest and teak plantations showed little or no phylogenetic structuring. In the natural forests three out of ten assemblages were significantly structured, with one plot significantly clustered (Plot B: NRI = 1.31, P < 0.05) and two assemblages significantly overdispersed (Plot E: NRI = -1.50; Plot L: NRI = -1.52, P < 0.05), whereas the teak plantations showed no phylogenetic structuring. Here NRI values did not deviate from random assemblages.

NRI values did not significantly differ between forest and teak plantation sites (Mann-Whitney-U test, Z = -0.87, N = 17, P = 0.417; Fig 3d), but species richness was significantly different between both habitat regimes with more species in the forest than teak plantations (Mann-Whitney-U test, Z = -2.31, N = 17, P = 0.019; Fig 3e). Also the number of total encounters was significantly higher in the forest sites than in the teak plantations (Mann-Whitney-U test, Z = -3.12, N = 17, P = 0.001; Fig 3f). Furthermore, there was a highly significant difference between the encounter rates of termites in the tropical forest and savannah sites, with many more termites encountered in the forest compared to the savannah (Mann-Whitney-U test, Z = -5.53, N = 44, P < 0.001).

### Similarity within and between habitat regimes

#### Savannah

**Fig 4:**
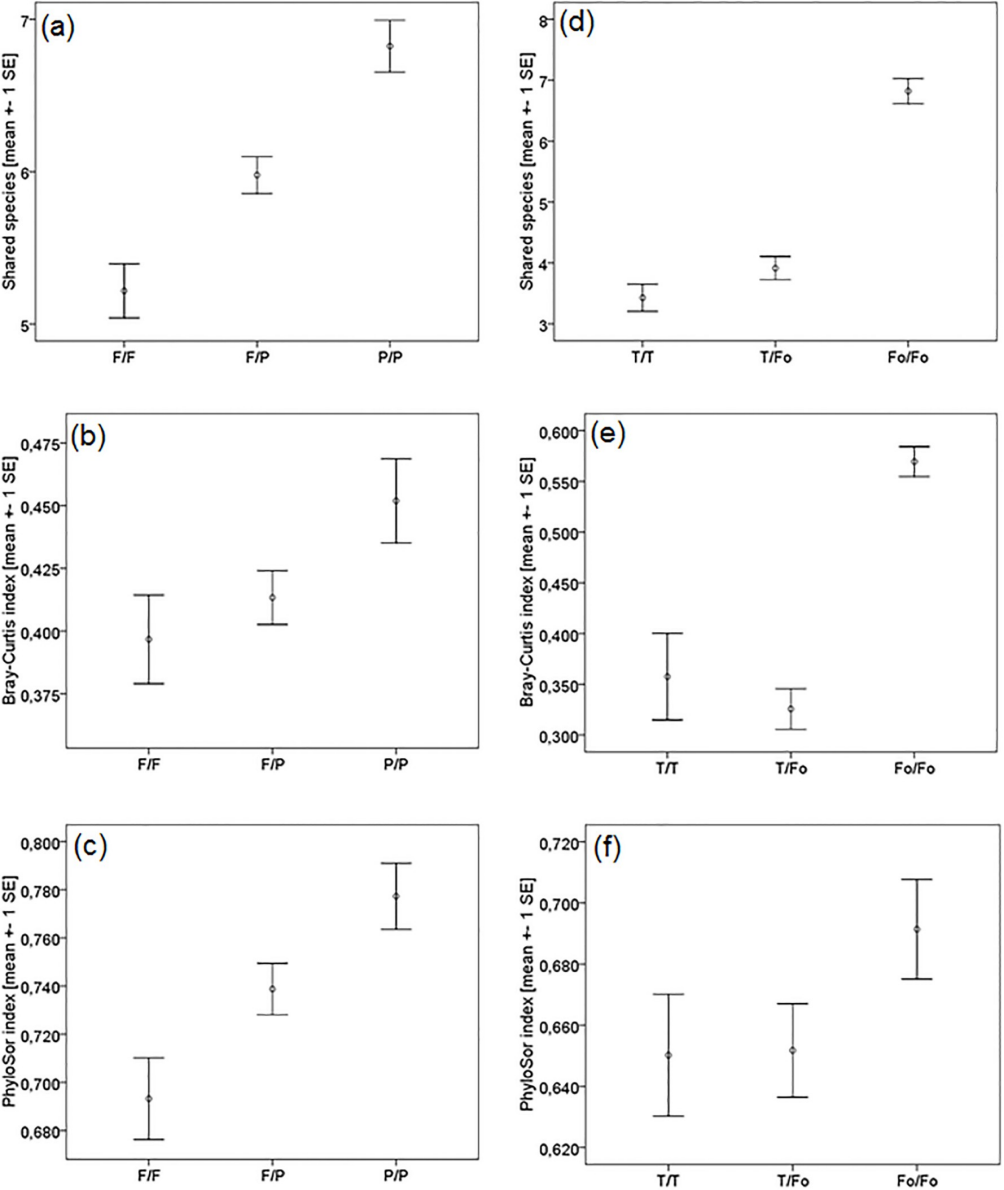
Similarity among sites within and between habitat regimes in the savannah (a-c) and forest (d-f) ecosystem. Figures (a) and (d) show the number of shared species, (b,e) compositional similarity measured with the Bray-Curtis index and (c,f) phylogenetic similarity measured with the PhyloSor index. F = fallows, P = Park; T = Teak plantation, Fo = Forest.

The compositional similarity varied between sites. The Bray-Curtis similarity index ranged from 0.029 to 0.772 for the protected Park and 0.108 to 0.785 for the fallows. The phylogenetic similarity between sites, measured with PhyloSor, varied from 0.145 to 0.981 in the Park and 0.145 to 0.951 in the fallows (S3 Table). Mean species richness per site was 10.6 (+- SD 1.93) in the Park and 10.1 (+- SD 1.75) in the fallows and mean number of shared species between sites was 6.8 (+- SD 1.62) in the Park and 5.2 (± SD 1.56) in the fallows. The number of shared species differed significantly when comparing sites within and between habitat regimes (ANOVA: F = 21.03, P < 0.001, Fig 4a). The number of shared species was higher among Park sites than in the fallows, with intermediate values when comparing Park with fallow sites. Similarly, the Bray-Curtis index and the PhyloSor index showed significant differences in compositional and phylogenetic similarity within and between habitat regimes (Bray-Curtis: ANOVA: F = 3.12, P = 0.045; PhyloSor: ANOVA: F = 7.31, P = 0.001; Fig 4b and 4c). Park sites seemed to consist of more similar species, compositionally and phylogenetically, compared to the fallows, where species—and phylogenetic composition was less similar between sites.

#### Forest

The compositional similarity between sites varied in the forest as well. In the protected forest the Bray-Curtis similarity index ranged from 0.086 to 0.781 and in the teak plantations from 0.111 to 0.687 (S4 Table). The PhyloSor index ranged from 0.303 to 0.899 in the forest and from 0.468 to 0.857 in the teak plantations. Mean species richness per site was 11.6 (+- SD 2.27) in the forest and 7.8 (+- SD 2.96) in the teak plantations. Mean number of shared species between sites was 6.8 (+- SD 1.38) in the forest and 3.4 (± SD 1.02) in the teak plantations. Therefore, the number of shared species differed significantly between protected forest sites and teak plantations (ANOVA: F = 65.84, P < 0.001; Fig 4d). Teak plantation sites shared significantly fewer species than the forest sites and also the number of shared species across plantation and forest sites was low. Compositional similarity, measured with the Bray-Curtis index, also significantly differed between the two habitat regimes, with the forest sites having a significantly higher compositional similarity than the teak plantations (ANOVA: F = 36.06, P < 0.001; Fig 4e). These results are in accordance with the results for the savannah, where disturbed sites shared fewer species and had a significantly lower compositional termite species similarity as well. Only the phylogenetic similarity of forest and teak plantations, measured with the PhyloSor index, did not differ significantly (ANOVA: F = 1.76, P = 0.176; Fig 4f), which was different in the savannah.

## Discussion

This is the first study that comparatively investigated forest and savannah ecosystems using the same means. We found striking similarities and differences between the forest and savannah ecosystems when analyzing protected and disturbed sites. For both ecosystems, we had hypothesized that disturbance negatively affects species richness. In neither of the two ecosystems did species richness change with time since disturbance. However, comparing disturbed and protected sites, species richness did decline in the forests while this was not the case for the savannah. As expected, we found in total more species in the forest than the savannah and also the termite encounter rates were higher in the former. Yet per site, species richness did not differ between both ecosystems. Finally, the similarity in termite composition between disturbed sites was low in both ecosystems, whereas it was high between protected sites. Yet, while species similarity between protected and disturbed sites was intermediate between fallow and protected savannahs sites, it was low between protected forests and teak plantations (Fig 4). Concerning assemblage structure, NRI values did not differ between disturbed and protected sites in both ecosystems and there were few signs of phylogenetic structuring. Finally, while the similarity in termite composition between protected and disturbed sites was intermediate between fallow and protected savannahs sites, it was low between protected forests and teak plantations (Fig 4). In both ecosystems, species similarity between disturbed sites was low, while it was high between protected sites.

### Savannah: Phylogenetic and compositional community structure

Our study revealed that the assemblages of the two studied savannah regimes differed significantly in their compositional (measured by shared species and with the Bray-Curtis index) and phylogenetic similarity (measured with the Phylosor index) (Fig 4a–4c). Assemblages in the Park are more similar to each other compositionally and phylogenetically than the ones in the fallows with intermediate similarities between both regimes (Fig 4a–4c). Park assemblages seem to have very similar species compositions in each sampled site, specific for this ecosystem. As one can assume assemblages in a National Park to be less disturbed, they have a long ‘assembly history’, with more or less stable abiotic and biotic factors. This is reflected in a medium-dense shrub savannah characterized by *Crossopteryx febrifuga*, *Vitellaria paradoxa*, *Pilliostigma thonningii*, *Afzelia africana*, *Combretum spp*. and *Terminalia spp*. (see S1 Fig). Disturbed assemblages seem to experience a higher turnover of species, resulting in an apparently less similar termite species composition, and more variable vegetation ranging from open fields via grassy savannahs to medium-dense shrub savannah (S1 Fig). Despite the fact that NRI values did not change with fallow age, our previous study showed that species composition actually differed depending on the respective age of the fallow [13]. Younger fallows had other species (*Amitermes evuncifer*, *Pericapritermes* sp., *Ancistrotermes* sp., *Microtermes* sp.*)* than older fallows, and these older fallows species were still different (*Pseudacanthotermes militaris*, *Fulleritermes tenebricus*) to those occurring in the Park, although the oldest fallows had an age of 12 years [13]. Therefore, comparing species composition of the different aged fallows can explain these intermediate similarities between Park and fallow sites.

### Comparison to similar studies

Our results partially correspond with a similar study in West Africa, looking at termite community assembly in Benin [10]. Here the impact of anthropogenic disturbance on termite assemblages in areas of intensive land-use was studied in comparison to assemblages in a National Park. As in our study, protected sites were similar to each other but differed in similarity to those from disturbed village sites. In contrast to our study in Togo, however, the disturbed sites in Benin were similar to each other and had very few termite species. This difference is in line with the much stronger degree of disturbance in Benin where disturbed study sites were next to villages and included active agricultural fields, compared to the fallows in our current study that lack on-going disturbance. In both studies, termite encounter rates were lower under disturbed than protected regimes. This also applied to our forest results and suggests that there is a general pattern: first species abundance declines with disturbance, and later with more intense disturbance, species numbers dwindle.

The decline of termite species richness with disturbance has been found in several studies across all continents (e.g. Ivory Coast: Coulibaly *et al*. [52]; Borneo: Luke *et al*. [53]; Vietnam: Neoh *et al*. [54] and Panama: Basset *et al*. [55]) supporting the hypothesis that it is a global pattern. Generally, the effect is more prominent in tropical forest regions than in savannahs.

### Forests: Phylogenetic and compositional community structure

Similar to the savannah, compositional and phylogenetic similarity in the forests were high among protected forest sites, but low between disturbed teak sites (Fig 4d–4f), probably for the same reasons as in the savannah that no or low disturbance in the protected sites lead to similar stable conditions and similar assemblages. In contrast to the savannah, however, the similarity between protected forest and teak plantations is low, while it was intermediate in the savannah. This is due to some changes in termite species composition, probably associated with vegetation changes (S2 Fig). In the teak plantations mainly Nasutitermitinae disappeared; three out of four *Trinervitermes* species were missing which are mainly grass-feeders [56]. Grass is largely missing in the teak mono-cultures, which are characterized by an open and plant-poor understory, while the protected forest was com posed by a high diversity of plants (S2 Fig).

### Comparison to similar studies

In other forest studies there was a notable decline of soil feeders with the transformation of forests into plantations and an increase in fungus growing Macrotermitinae [57,58]. Unexpectedly, this was not the case in our study (Table 1). Two Macrotermitinae (*M*. *bellicosus* and one *Odontotermes* species) disappeared in the plantations but one fungus grower, *Pseudacanthotermes militaris*, was only found in the plantations. In general, there were few true soil- (feeding group IV; four species) and humus-feeders (feeding group III; five species) in our study. Three true soil feeders occurred in both forest types, while the plantations had all five humus feeders but one was missing from the protected forest.

These differences in the effect of disturbance on termite assemblage composition compared to other forest studies might be due to regional differences. Our species richness was rather low compared to tropical forests in central Africa or America, which have many more soil- and humus feeders, especially Apicotermitinae. The fact that teak plantations are still forests (S2 Fig), and not open areas like fallows or cultivated fields, may explain why we did not see an increase in Macrotermitinae with disturbance.

### Comparing forests and savannahs

Comparing forests and savannahs, both ecosystems differed in the number of soil feeders with *Basidentitermes*, *Proboscitermes* and *Lepidotermes* only occurring in the former. The single soil feeder found in the savannah was a *Noditermes* sp. Correspondingly, the higher species richness in the forest was mainly due to more Termitinae. In addition, two lower termites occurred in the forest while the savannah had only higher termites (Table 1). The number of humus-feeding termites, fungus-growing termites and grass feeders were similar in both ecosystems, yet species overlap was restricted for the first two. *Procubitermes* sp. were savannah-specific while we found *Aderitotermes fossor* only in the forest. For fungus growers, except for *Macrotermes*, the species repertoire also differed between both ecosystems. Strikingly, a completely different set of *Ancistrotermes* and partly also *Microtermes* species were identified for the savannah and the forest, with only a few of the described species occurring in the savannah (Table 1). Here, it is important to note the difficulties in delimitation *A*. *guineensis* and *A*. *crucifer* as single species in the forest. This implies that more studies are needed to confirm or reject the species status of both species. As species from these genera are extremely difficult to distinguish morphologically (Table 1), these species might often have been misclassified in other studies.

Overall, we found six species that occurred in all four studied habitats: *Microtermes lepidus*, *Microtermes subhyalinus*, *Astalotermes quietus*, *Adaiphrotermes* sp., *Microcerotermes* sp.1 and *Amitermes evuncifer*. *Microtermes*, *Adaiphrotermes*, *Microcerotermes* and *A*. *evuncifer* have been associated with disturbance [13] and are important pests. *A*. *evuncifer* causes considerable damage in teak plantations [59]. This widespread occurrence implies that they are generalists with low habitat requirements.

Comparing NRI values, we found less evidence for phylogenetic structuring of termite assemblages. Niche traits like feeding type are phylogenetically conserved in termites [13], so that phylogenetic overdispersion would indicate to interspecific competition and phylogenetic clustering to environmental filtering. The community of some plots differed from random assemblages but not consistently across ecosystems or disturbance regimes. Also we did not find evidence that the structuring mechanisms change with the time since last disturbance.

To summarize, our study reliably identified a total of 40 termite species, covering the two major West African ecosystems, savannah and forest, including natural as well as disturbed sites. Thus, this study can serve as the basis for upcoming ecological research which relies on proper and exact species identification. Although we found in total more species in the forests than in the savannah, the local species richness was with a mean of around 10 species not different. The species repertoire, especially of fungus growers, differed greatly between both ecosystems but we also identified several generalist species. We have no evidence that the termite assemblages structuring mechanisms differ systematically from random combinations of the regional species pool. Disturbance rather generally seems to lead first to a decline in termite abundance and then in species richness. Given the importance that termites play as ecosystem engineers, this implies declining ecosystem services with increasing disturbance.

## Supporting information

S1 Fig Vegetation of savannah study area. (a) Protected site located in the Oti-Kéran National Park, (b) 1-year old fallow, (c) 4-year old fallow, and (d) 12 year old fallow. J. Schyra.
(PDF)

S2 Fig Vegetation of forest study area. (a) Protected site located in the Reserve de Faune de Togodo, (b) 2-year old teak plantation, (c) 6-year old teak plantation, and (d) 12-year old teak plantation. J.N. Gbenyedji.
(PDF)

S3 Fig Best maximum likelihood tree for the savannah with bootstrap support. (PDF)

S4 Fig Best maximum likelihood tree for the forest with bootstrap support. (PDF)

S5 Fig Results of species delimitation approach for savannah dataset. Shown is a tree with all analysed samples. Green lines support delimitation of separate species, samples linked by red lines are not delimited as separate species.
(PDF)

S6 Fig Results of species delimitation approach for forest dataset. Shown is a tree with all analysed samples. Green lines support delimitation of separate species, samples linked by red lines are not delimited as separate species.
(PDF)

S1 Table Genbank accession numbers for savannah dataset. (TXT)

S2 Table Genbank accession numbers for forest dataset. (TXT)

S3 Table ß-diversity and phylogenetic ß-diversity indices for all study plot pairs in the natural and disturbed habitat regimes in the savannah. (PDF)

S4 Table ß-diversity and phylogenetic ß-diversity indices for all study plot pairs in the protected and disturbed habitat regimes in the forest ecosystem. (PDF)

S1 Appendix Alignment used for species delimitation of savannah dataset. (FAS)

S2 Appendix Alignment used for species delimitation of forest dataset. (FAS)

S3 Appendix Results of species delimitation approach for savannah dataset. (TXT)

S4 Appendix Results of species delimitation approach for forest dataset. (TXT)
