# Supplementary material for: A comparison of termite assemblages from West African savannah and forest ecosystems using morphological and molecular markers
Source: PLoS One. 2019 Jun 5;14(6):e0216986. doi: 10.1371/journal.pone.0216986 (PMC6550446; doi:10.1371/journal.pone.0216986)

S1 Fig Vegetation of savannah study area. (a) Protected site located in the Oti-Kéran National Park, (b) 1-year old fallow, (c) 4-year old fallow, and (d) 12 year old fallow. © J. Schyra

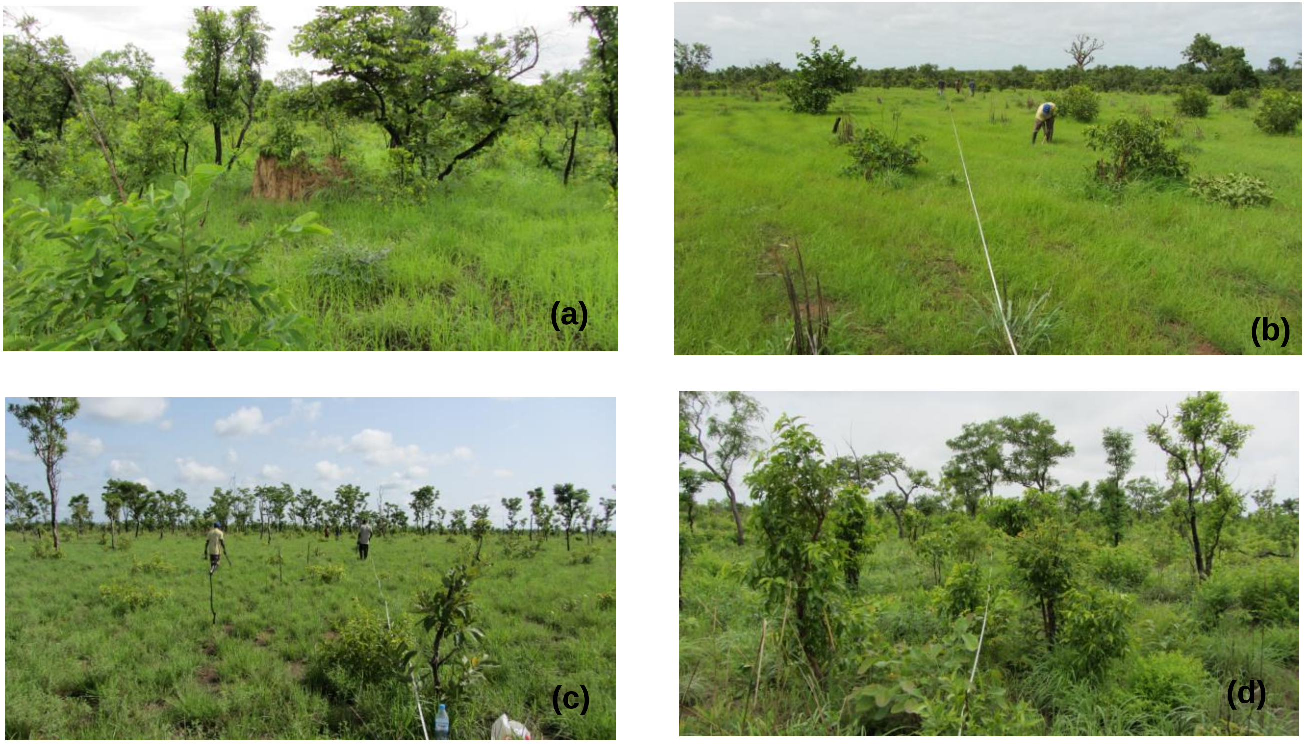

Supplement: S1 Fig — (a) Protected site located in the Oti-Kéran National Park, (b) 1-year old fallow, (c) 4-year old fallow, and (d) 12 year old fallow. J. Schyra. (PDF) [file pone.0216986.s001.pdf]
