# Supplementary material for: A comparison of termite assemblages from West African savannah and forest ecosystems using morphological and molecular markers
Source: PLoS One. 2019 Jun 5;14(6):e0216986. doi: 10.1371/journal.pone.0216986 (PMC6550446; doi:10.1371/journal.pone.0216986)

S2 Fig Vegetation of forest study area. (a) Protected site located in the Reserve de Faune de Togodo, (b) 2-year old teak plantation, (c) 6-year old teak plantation, and (d) 12-year old teak plantation. © J.N. Gbenyedji.

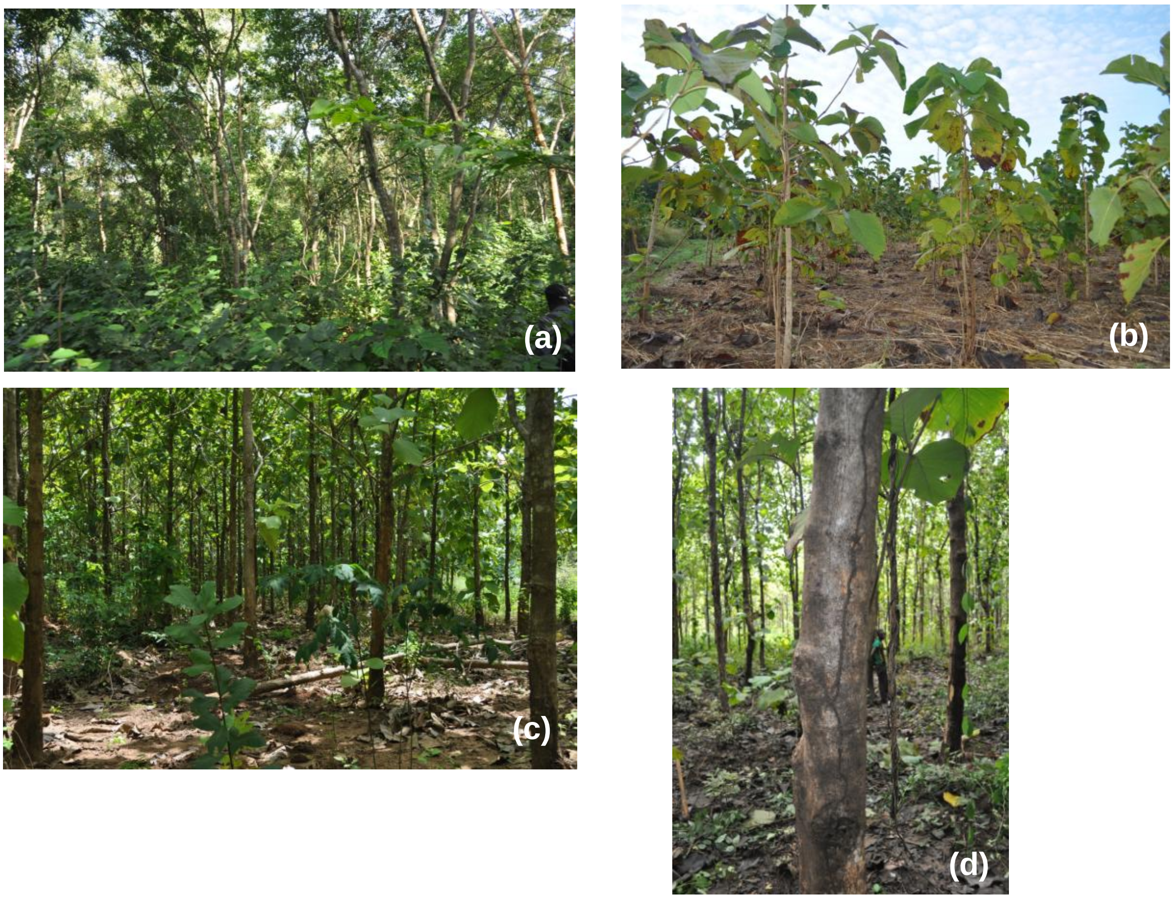

Supplement: S2 Fig — (a) Protected site located in the Reserve de Faune de Togodo, (b) 2-year old teak plantation, (c) 6-year old teak plantation, and (d) 12-year old teak plantation. J.N. Gbenyedji. (PDF) [file pone.0216986.s002.pdf]
