# Supplementary figures and images for: A comparison of termite assemblages from West African savannah and forest ecosystems using morphological and molecular markers
Source: PLoS One. 2019 Jun 5;14(6):e0216986. doi: 10.1371/journal.pone.0216986 (PMC6550446; doi:10.1371/journal.pone.0216986)

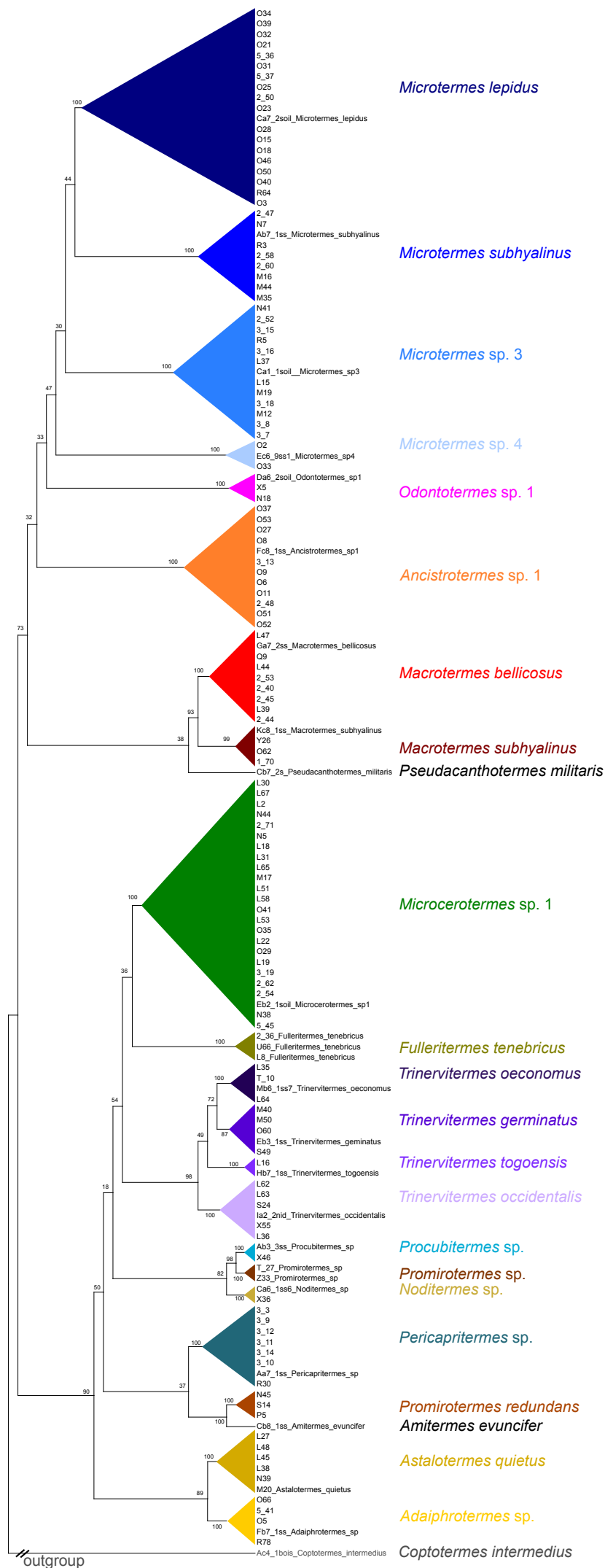

Supplement: S3 Fig — (PDF) [file pone.0216986.s003.pdf]

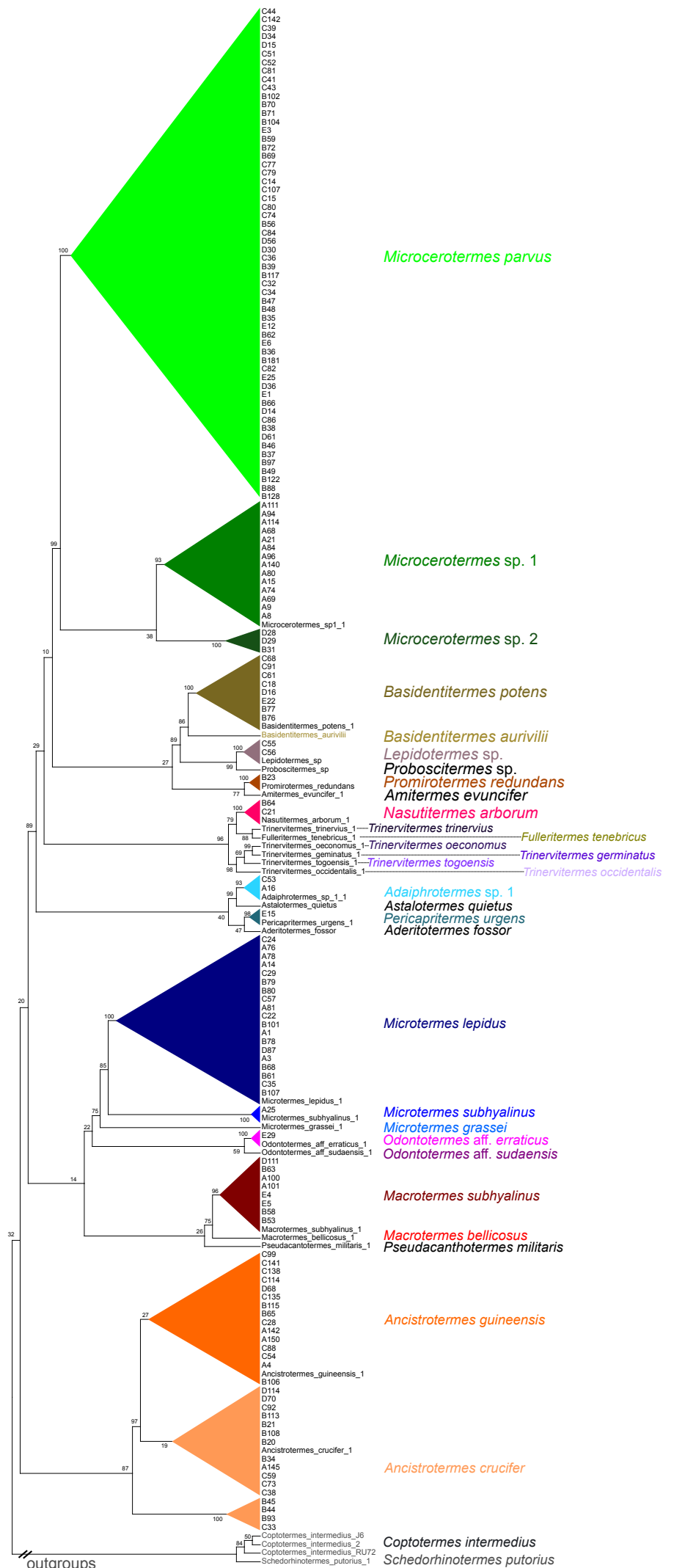

Supplement: S4 Fig — (PDF) [file pone.0216986.s004.pdf]

outgroup

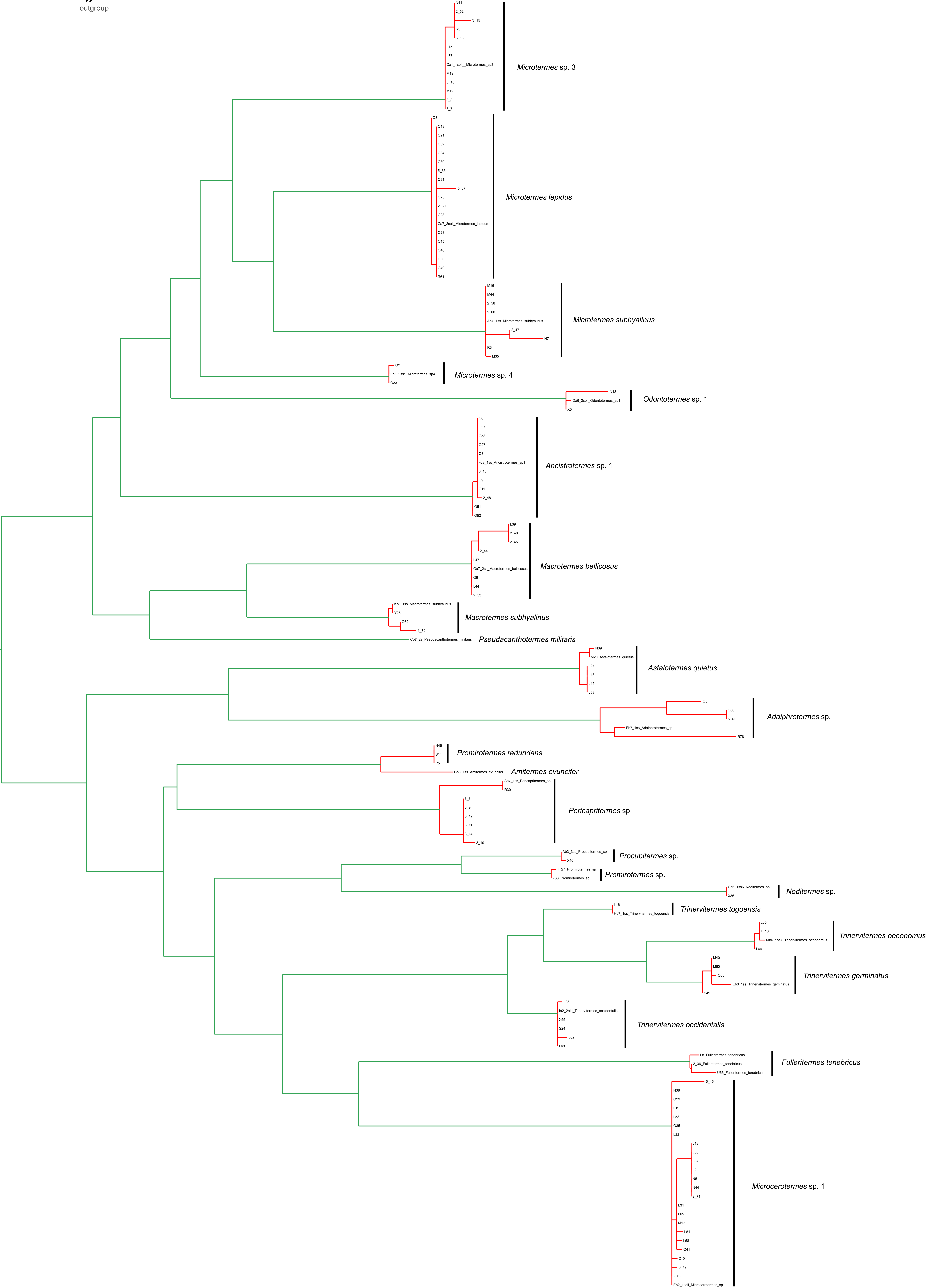

Supplement: S5 Fig — Shown is a tree with all analysed samples. Green lines support delimitation of separate species, samples linked by red lines are not delimited as separate species. (PDF) [file pone.0216986.s005.pdf]

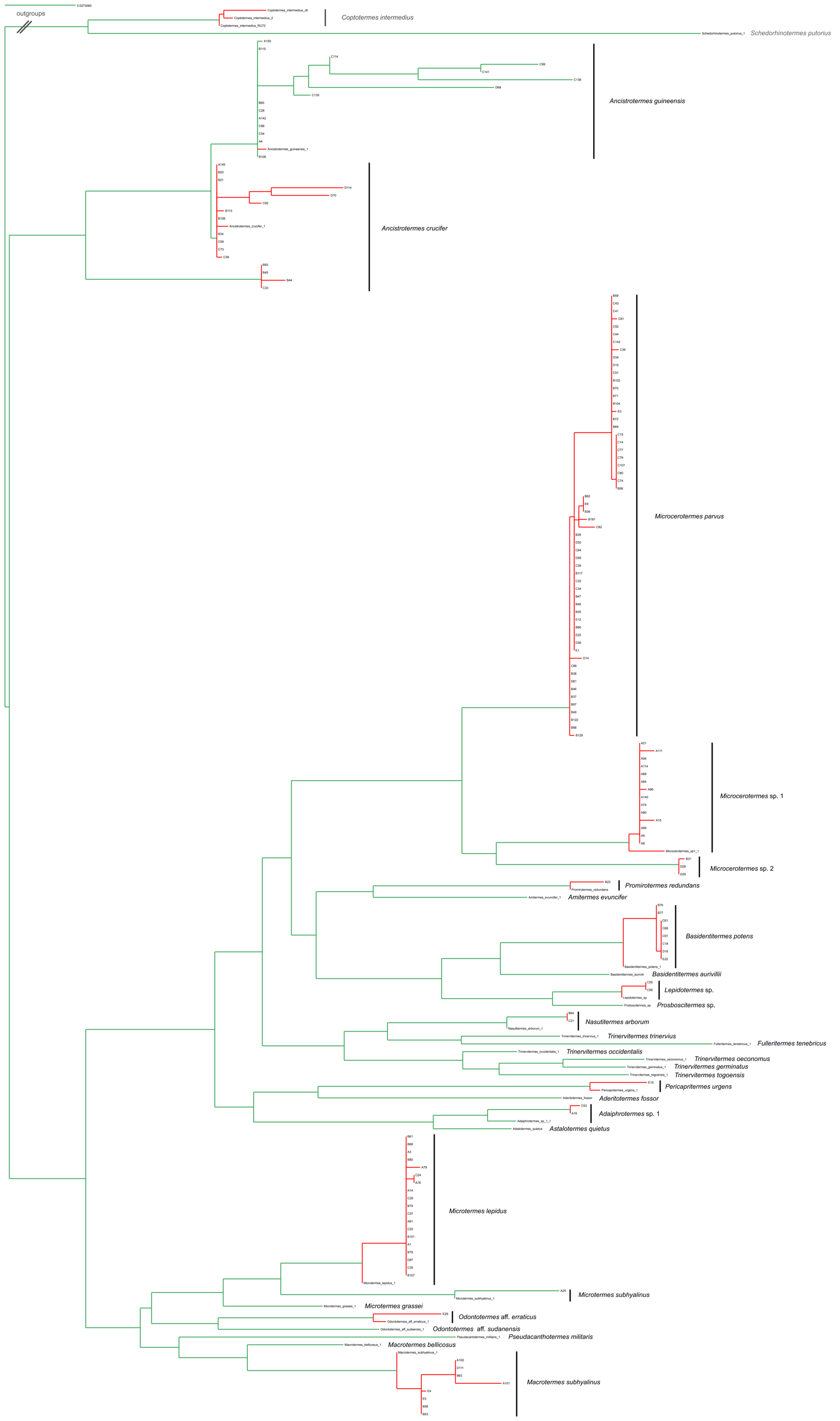

Supplement: S6 Fig — Shown is a tree with all analysed samples. Green lines support delimitation of separate species, samples linked by red lines are not delimited as separate species. (PDF) [file pone.0216986.s006.pdf]
