# Supplementary material for: A comparison of termite assemblages from West African savannah and forest ecosystems using morphological and molecular markers
Source: PLoS One. 2019 Jun 5;14(6):e0216986. doi: 10.1371/journal.pone.0216986 (PMC6550446; doi:10.1371/journal.pone.0216986)
Supplement: S3 Table — (PDF) [file pone.0216986.s009.pdf]

S 3Table ß-diversity and phylogenetic ß-diversity indices for all study plot pairs in the natural and disturbed habitat regimes in the savannah.

Plot 1	Plot 2	Bray-Curtis	PhyloSor
L	M	0.677	0.762
L	N	0.451	0.718
L	O	0.193	0.605
L	P	0.569	0.672
L	R	0.643	0.730
L	S	0.644	0.687
L	T	0.441	0.722
L	U	0.596	0.868
L	W	0.122	0.387
L	2	0.391	0.887
L	3	0.367	0.758
L	5	0.115	0.496
L	B	0.503	0.764
L	C	0.275	0.741
L	D	0.591	0.726
L	E	0.475	0.697
L	F	0.562	0.798
L	G	0.496	0.779
L	H	0.491	0.706
L	I	0.36	0.672
L	K	0.569	0.811
L	Q	0.5	0.838
L	X	0.463	0.763
L	Y	0.512	0.720
L	Z	0.483	0.870
L	1	0.126	0.496
M	N	0.424	0.842
M	O	0.225	0.738
M	P	0.566	0.729
M	R	0.613	0.951
M	S	0.417	0.812
M	T	0.316	0.775
M	U	0.507	0.621
M	W	0.172	0.588
M	2	0.267	0.748
M	3	0.264	0.821
M	5	0.101	0.503

M	B	0.473	0.893
M	C	0.28	0.900
M	D	0.393	0.872
M	E	0.336	0.833
M	F	0.322	0.865
M	G	0.279	0.769
M	H	0.272	0.763
M	I	0.474	0.729
M	K	0.346	0.718
M	Q	0.416	0.897
M	X	0.382	0.922
M	Y	0.41	0.817
M	Z	0.342	0.775
M	1	0.129	0.503
N	O	0.288	0.771
N	P	0.427	0.666
N	R	0.453	0.800
N	S	0.429	0.758
N	T	0.529	0.918
N	U	0.542	0.681
N	W	0.268	0.627
N	2	0.385	0.722
N	3	0.427	0.771
N	5	0.159	0.533
N	B	0.41	0.845
N	C	0.202	0.750
N	D	0.593	0.844
N	E	0.477	0.805
N	F	0.393	0.818
N	G	0.33	0.621
N	H	0.389	0.708
N	I	0.303	0.666
N	K	0.542	0.780
N	Q	0.6	0.749
N	X	0.476	0.775
N	Y	0.527	0.662
N	Z	0.349	0.729
N	1	0.171	0.533
O	P	0.169	0.570
O	R	0.362	0.793
O	S	0.22	0.756
O	T	0.303	0.803
O	U	0.413	0.736

O	W	0.6	0.643
O	2	0.653	0.767
O	3	0.39	0.667
O	5	0.473	0.712
O	B	0.375	0.733
O	C	0.321	0.749
O	D	0.306	0.833
O	E	0.321	0.931
O	F	0.335	0.712
O	G	0.373	0.778
O	H	0.348	0.712
O	I	0.073	0.570
O	K	0.302	0.822
O	Q	0.248	0.716
O	X	0.331	0.771
O	Y	0.239	0.828
O	Z	0.357	0.692
O	1	0.514	0.712
P	R	0.496	0.688
P	S	0.549	0.798
P	T	0.404	0.677
P	U	0.509	0.643
P	W	0.212	0.175
P	2	0.252	0.576
P	3	0.366	0.724
P	5	0.042	0.145
P	B	0.452	0.733
P	C	0.413	0.703
P	D	0.523	0.687
P	E	0.379	0.589
P	F	0.487	0.780
P	G	0.388	0.675
P	H	0.329	0.822
P	I	0.63	1.000
P	K	0.408	0.628
P	Q	0.468	0.632
P	X	0.451	0.734
P	Y	0.469	0.726
P	Z	0.43	0.682
P	1	0.122	0.145
R	S	0.557	0.874
R	T	0.5	0.832
R	U	0.593	0.676

R	W	0.311	0.666
R	2	0.394	0.794
R	3	0.513	0.879
R	5	0.222	0.574
R	B	0.568	0.947
R	C	0.331	0.951
R	D	0.588	0.920
R	E	0.556	0.880
R	F	0.493	0.919
R	G	0.527	0.824
R	H	0.527	0.826
R	I	0.269	0.688
R	K	0.537	0.769
R	Q	0.481	0.857
R	X	0.561	0.971
R	Y	0.481	0.780
R	Z	0.516	0.829
R	1	0.213	0.574
S	T	0.593	0.797
S	U	0.488	0.749
S	W	0.177	0.461
S	2	0.3	0.676
S	3	0.462	0.848
S	5	0.058	0.389
S	B	0.586	0.926
S	C	0.281	0.820
S	D	0.68	0.796
S	E	0.596	0.758
S	F	0.625	0.894
S	G	0.654	0.790
S	H	0.682	0.943
S	I	0.341	0.798
S	K	0.518	0.733
S	Q	0.535	0.717
S	X	0.479	0.846
S	Y	0.654	0.738
S	Z	0.471	0.796
S	1	0.128	0.389
T	U	0.61	0.768
T	W	0.321	0.680
T	2	0.319	0.798
T	3	0.44	0.869
T	5	0.197	0.587

T	B	0.636	0.875
T	C	0.248	0.843
T	D	0.661	0.924
T	E	0.557	0.833
T	F	0.589	0.909
T	G	0.704	0.720
T	H	0.642	0.816
T	I	0.28	0.677
T	K	0.446	0.858
T	Q	0.346	0.693
T	X	0.475	0.865
T	Y	0.64	0.671
T	Z	0.483	0.820
T	1	0.161	0.587
U	W	0.338	0.455
U	2	0.462	0.920
U	3	0.474	0.700
U	5	0.331	0.552
U	B	0.615	0.707
U	C	0.304	0.687
U	D	0.5	0.769
U	E	0.394	0.739
U	F	0.517	0.741
U	G	0.523	0.809
U	H	0.505	0.766
U	I	0.343	0.643
U	K	0.504	0.848
U	Q	0.422	0.702
U	X	0.509	0.709
U	Y	0.523	0.673
U	Z	0.544	0.813
U	1	0.212	0.552
W	2	0.496	0.613
W	3	0.442	0.586
W	5	0.53	0.833
W	B	0.301	0.582
W	C	0.446	0.659
W	D	0.345	0.728
W	E	0.462	0.691
W	F	0.355	0.561
W	G	0.333	0.520
W	H	0.257	0.452
W	I	0.063	0.175

W	K	0.256	0.568
W	Q	0.242	0.541
W	X	0.374	0.644
W	Y	0.233	0.438
W	Z	0.365	0.517
W	1	0.426	0.833
2	3	0.577	0.738
2	5	0.5	0.683
2	B	0.275	0.744
2	C	0.452	0.803
2	D	0.444	0.871
2	E	0.419	0.840
2	F	0.446	0.774
2	G	0.441	0.835
2	H	0.352	0.692
2	I	0.118	0.576
2	K	0.515	0.868
2	Q	0.453	0.815
2	X	0.481	0.821
2	Y	0.362	0.711
2	Z	0.557	0.839
2	1	0.568	0.683
3	5	0.337	0.500
3	B	0.339	0.927
3	C	0.46	0.931
3	D	0.591	0.862
3	E	0.547	0.768
3	F	0.588	0.962
3	G	0.612	0.798
3	H	0.532	0.903
3	I	0.137	0.724
3	K	0.485	0.797
3	Q	0.519	0.762
3	X	0.571	0.912
3	Y	0.408	0.740
3	Z	0.645	0.895
3	1	0.34	0.500
5	B	0.19	0.497
5	C	0.302	0.568
5	D	0.198	0.635
5	E	0.222	0.749
5	F	0.227	0.482
5	G	0.306	0.617

5	H	0.217	0.382
5	I	0.023	0.145
5	K	0.328	0.736
5	Q	0.2	0.638
5	X	0.26	0.557
5	Y	0.108	0.540
5	Z	0.358	0.615
5	1	0.6	1.0
B	C	0.262	0.896
B	D	0.538	0.868
B	E	0.453	0.829
B	F	0.534	0.968
B	G	0.571	0.765
B	H	0.579	0.874
B	I	0.365	0.733
B	K	0.441	0.803
B	Q	0.353	0.798
B	X	0.48	0.919
B	Y	0.571	0.715
B	Z	0.474	0.870
B	1	0.116	0.497
C	D	0.333	0.929
C	E	0.36	0.838
C	F	0.491	0.929
C	G	0.366	0.872
C	H	0.244	0.839
C	I	0.325	0.703
C	K	0.422	0.779
C	Q	0.347	0.811
C	X	0.633	0.981
C	Y	0.338	0.791
C	Z	0.496	0.840
C	1	0.304	0.568
D	E	0.772	0.911
D	F	0.64	0.899
D	G	0.692	0.813
D	H	0.682	0.813
D	I	0.304	0.687
D	K	0.642	0.853
D	Q	0.53	0.790
D	X	0.561	0.948
D	Y	0.654	0.772
D	Z	0.586	0.818

D	1	0.209	0.635
E	F	0.576	0.807
E	G	0.595	0.778
E	H	0.609	0.718
E	I	0.186	0.589
E	K	0.552	0.896
E	Q	0.489	0.806
E	X	0.479	0.858
E	Y	0.559	0.825
E	Z	0.528	0.783
E	1	0.238	0.749
F	G	0.696	0.801
F	H	0.569	0.912
F	I	0.309	0.780
F	K	0.5	0.833
F	Q	0.474	0.775
F	X	0.647	0.950
F	Y	0.563	0.754
F	Z	0.6	0.903
F	1	0.228	0.482
G	H	0.716	0.808
G	I	0.202	0.675
G	K	0.521	0.814
G	Q	0.43	0.849
G	X	0.497	0.856
G	Y	0.579	0.821
G	Z	0.661	0.876
G	1	0.258	0.617
H	I	0.2	0.822
H	K	0.5	0.750
H	Q	0.432	0.706
H	X	0.4	0.863
H	Y	0.632	0.792
H	Z	0.6	0.846
H	1	0.139	0.382
I	K	0.383	0.628
I	Q	0.294	0.632
I	X	0.387	0.734
I	Y	0.337	0.726
I	Z	0.286	0.682
I	1	0.029	0.145
K	Q	0.531	0.792
K	X	0.571	0.799

K	Y	0.471	0.765
K	Z	0.579	0.900
K	1	0.274	0.736
Q	X	0.484	0.833
Q	Y	0.452	0.819
Q	Z	0.568	0.882
Q	1	0.239	0.638
X	Y	0.55	0.815
X	Z	0.597	0.861
X	1	0.273	0.557
Y	Z	0.55	0.760
Y	1	0.147	0.540
Z	1	0.291	0.615
