# Supplementary material for: A comparison of termite assemblages from West African savannah and forest ecosystems using morphological and molecular markers
Source: PLoS One. 2019 Jun 5;14(6):e0216986. doi: 10.1371/journal.pone.0216986 (PMC6550446; doi:10.1371/journal.pone.0216986)
Supplement: S4 Table — (PDF) [file pone.0216986.s010.pdf]

S4 Table. ß-diversity and phylogenetic ß-diversity indices for all study plot pairs in the protected and disturbed habitat regimes in the forest ecosystem.

Plot 1	Plot 2	Bray-Curtis	PhyloSor
A	B	0.587	0.632
A	C	0.52	0.603
A	D	0.441	0.703
A	E	0.408	0.634
A	H	0.588	0.745
A	I	0.412	0.697
A	K	0.489	0.676
A	L	0.494	0.583
A	M	0.553	0.624
A	O	0.358	0.518
A	R	0.406	0.665
A	S	0.705	0.601
A	P	0.419	0.497
A	G	0.631	0.79
A	F	0.363	0.386
A	T	0.279	0.664
B	C	0.708	0.832
B	D	0.465	0.599
B	E	0.463	0.552
B	H	0.546	0.849
B	I	0.611	0.87
B	K	0.59	0.704
B	L	0.662	0.846
B	M	0.59	0.899
B	O	0.235	0.801
B	R	0.277	0.811
B	S	0.566	0.677
B	P	0.488	0.641
B	G	0.56	0.653
B	F	0.198	0.664
B	T	0.169	0.827
C	D	0.609	0.57
C	E	0.545	0.53
C	H	0.594	0.702
C	I	0.674	0.725
C	K	0.557	0.681
C	L	0.59	0.7
C	M	0.587	0.75
C	O	0.155	0.647
C	R	0.188	0.783
C	S	0.486	0.524
C	P	0.466	0.648
C	D	0.426	0.549
C	F	0.248	0.671
C	T	0.183	0.694
D	E	0.482	0.685
D	H	0.665	0.629
D	I	0.534	0.544
D	K	0.481	0.652
D	L	0.478	0.527

D	M	0.608	0.529
D	O	0.135	0.483
D	R	0.132	0.607
D	S	0.417	0.576
D	P	0.379	0.303
D	G	0.426	0.613
D	F	0.188	0.32
D	T	0.14	0.501
E	H	0.492	0.603
E	I	0.464	0.582
E	K	0.419	0.745
E	L	0.454	0.552
E	M	0.417	0.527
E	O	0.173	0.518
E	R	0.184	0.612
E	S	0.367	0.484
E	P	0.434	0.481
E	G	0.376	0.59
E	F	0.249	0.404
E	T	0.138	0.584
H	I	0.616	0.812
H	K	0.581	0.755
H	L	0.632	0.821
H	M	0.659	0.838
H	O	0.086	0.706
H	R	0.163	0.758
H	S	0.445	0.657
H	P	0.376	0.617
H	G	0.469	0.765
H	F	0.251	0.638
H	T	0.145	0.748
I	K	0.724	0.658
I	L	0.685	0.809
I	M	0.722	0.859
I	O	0.101	0.793
I	R	0.154	0.843
I	S	0.595	0.720
I	P	0.461	0.689
I	G	0.495	0.680
I	F	0.172	0.630
I	T	0.145	0.768
K	L	0.73	0.699
K	M	0.72	0.745
K	O	0.091	0.734
K	R	0.148	0.801
K	S	0.613	0.607
K	P	0.425	0.510
K	G	0.498	0.594
K	F	0.2	0.549
K	T	0.152	0.702
L	M	0.781	0.836
L	O	0.114	0.704
L	R	0.191	0.756
L	S	0.588	0.842
L	P	0.461	0.615
L	G	0.536	0.612
L	F	0.265	0.844

L	T	0.253	0.746
M	O	0.162	0.851
M	R	0.256	0.884
M	S	0.678	0.669
M	P	0.442	0.632
M	G	0.577	0.628
M	F	0.278	0.654
M	T	0.269	0.890
O	R	0.580	0.857
O	S	0.384	0.662
O	P	0.336	0.628
O	G	0.687	0.594
O	F	0.118	0.520
O	T	0.667	0.790
R	S	0.612	0.671
R	P	0.132	0.639
R	G	0.445	0.634
R	F	0.181	0.583
R	T	0.575	0.796
S	P	0.204	0.562
S	G	0.335	0.680
S	F	0.180	0.655
S	T	0.424	0.595
P	G	0.469	0.671
P	F	0.268	0.725
P	T	0.119	0.678
G	F	0.178	0.468
G	T	0.504	0.671
F	T	0.111	0.575
